# Artificial Intelligence-Supported Ultrasonography in Anesthesiology: Evaluation of a Patient in the Operating Theatre

**DOI:** 10.3390/jpm14030310

**Published:** 2024-03-15

**Authors:** Sławomir Mika, Wojciech Gola, Monika Gil-Mika, Mateusz Wilk, Hanna Misiołek

**Affiliations:** 1 Medica Co., Ltd. (Upper Silesian School of Ultrasonography), 41-500 Chorzów, Poland; 2 Collegium Medicum, Jan Kochanowski University, 25-317 Kielce, Poland; wojciech.gola@ujk.edu.pl; 3 Municipal Hospital Co., Ltd., 41-703 Ruda Śląska, Poland; mmika@szpitalruda.pl; 4 Collegium Medicum, WSB University, 41-300 Dąbrowa Górnicza, Poland; mateusz.wilk@wsb.edu.pl; 5 Department of Anaesthesiology and Critical Care, School of Medicine with the Division of Dentistry, Medical University of Silesia, 41-808 Zabrze, Poland; hanna.misiolek@sum.edu.pl

**Keywords:** artificial intelligence, ultrasonography, regional anesthesia

## Abstract

Artificial intelligence has now changed regional anesthesia, facilitating, therefore, the application of the regional block under the USG guidance. Innovative technological solutions make it possible to highlight specific anatomical structures in the USG image in real time, as needed for regional block. This contribution presents such technological solutions as U-Net architecture, BPSegData and Nerveblox and the basis for independent assisting systems in the use of regional blocks, e.g., ScanNav Anatomy PNB or the training system NeedleTrainer. The article describes also the systems integrated with the USG devices, such as Mindray SmartNerve or GE cNerve as well as the robotic system Magellan which substantially increases the patient’s safety, time needed for the regional block and quality of the procedure. All the solutions presented in this article facilitate the performance of regional blocks by less experienced physicians and appear as an excellent educational tool which, at the same time, improves the availability of the more and more popular regional anesthesia. Will, therefore, artificial intelligence replace physicians in regional block procedures? This seems unlikely. It will, however, assist them in a significant manner, contributing to better effectiveness and improved safety of the patient.

## 1. Introduction

### 1.1. The Emergence of Artificial Intelligence in Medicine

Witnessing the fourth industrial revolution, we observe the characteristic blurring of the boundaries between physics, information technologies and biology which, in consequence, changes mankind, our lifestyles and working routines [1]. All these are related to the omnipresent artificial intelligence (AI) which is defined, in the simplest way, as the application of a computer to model intelligent behaviors with the minimal intervention of a human. It has been accepted that the idea emerged along with the invention of the very first robots, officially dated 1956 [2]. The electronic storage of medical data integrated with state-of-the-art computers managing the databases creates the informatics systems ensuring the specific nature of artificial intelligence developing in medicine. It is expected that modern technologies, including IT solutions, will contribute to 15% of the British GNP by 2030 [3]. So far, artificial intelligence has proved its applicability in such areas of medicine as the development of new drugs, diagnostic imaging, surgery, anesthesiology, intensive care as well as pain management. It is essential that this technological transformation is accomplished always under the control of a human [4].

### 1.2. AI in Anesthesiology—Objectives and Challenges

In particular, the use of AI in anesthesiology has been frequently discussed in both the literature and media throughout the recent two or three years. Consequently, the innovative technologies have resulted in the progressive development of medical devices dedicated to anesthesiologists [5]. Specifically, regional anesthesia offers a great potential for artificial intelligence to expand. The major objective for the use of AI in this area is to reduce the incidence of complications associated mainly with the undesirable puncture of a nerve, vessel, pleura or peritoneum, the optimization and interpretation of the image along with a visualization of the needle as well as an understanding of functions or the devices employed for the procedure [6]. Today, one could hardly imagine regional anesthesia without ultrasound devices enhancing the effectivity of the regional block performed [7]. Most often quoted in the literature are such indices of efficiency of the USG-assisted regional anesthetic procedures: improved effectivity of the block, reduced volume of the local anesthetic, monitoring of the local anesthetic diffusion, easy identification of the anatomical structures and of the needle position, reduced frequency of conversion to general anesthesia, substantially reduced duration of the procedure, smaller incidence of complications and quicker accomplishment of the optimum block [8]. Both the guidelines of the European Society of Anesthesiology and publications in the British Journal of Anesthesia point clearly to ultrasonography as the “golden standard” in the use of the regional block [9].

### 1.3. The Role of Ultrasound and AI in Anesthesia as an Assistive Tool in Clinical Practice

Guidance provided by the AI-assisted USG image may appear as one of the ways to improve effectiveness of the regional block [10]. A condition required to exploit the potential of the ultrasound technologies is the excellent command of anatomy shown by the operator. Unfortunately, graduates of medical schools still show insufficient expertise in the area of classical anatomy, topographic anatomy as well as sonoanatomy. Considerable individual anatomical diversity and frequently difficult sonoanatomical conditions may discourage less experienced anesthesiologists [11]. A skill indispensable in regional anesthesia to use ultrasonography effectively throughout the daily routines is the knowledge of the location of particular dermatomes, osteotomes and myotomes and understanding the mechanisms underlying individual regional blocks [12].

Within such a complicated environment, clinicists are offered assistance from artificial intelligence. Depending on the type of the software, it assists the detection of key structures for a given regional block. It may offer substantial help to identify not only the nerves but also the surrounding structures, such as blood vessels, muscles and fascia and bones guiding the needle through the tissues and pointing where the local anesthetic should be deposited.

## 2. Artificial Intelligence in Ultrasonography and Its Use in Regional

The identification of the neural structures, particularly by inexperienced trainees of regional anesthesiology, may appear a major problem. As pointed out earlier, the knowledge of sonoanatomy is a condition needed and indispensable to use ultrasonography in regional block procedures, the popularity of which is growing each year [11]. It has also been observed that the interpretation of AI-supported images may be one of the methods to improve the success rate for the procedure of nerves or nerve plexus blocks [10]. The “head-up-display” which shows the prompts in real time coding different anatomical structures with specific colors may ensure a shorter time needed to identify the neural structures and to confirm that further decisions made by the operator are correct [13]. The technology is now rapidly developing, including software capable not only of the recognition of anatomical structures but also tracing the needle in human tissues.

### 2.1. U-Net Architecture and BPSegData

Discussions on the recognition of anatomical structures by USG systems comprising a computer with appropriate software and peripheries, i.e., ultrasound heads, must not miss the notions of machine learning (ML) and deep learning (DL) and, in particular, deep convolutional neural networks (DCNNs). To make things simple, the notions refer to the commonly available IT tools which were originally developed as mathematical concepts. One of those, used earlier for the segmentation of biomedical images and later in medicine, is the U-Net architecture developed in 2015 by Olaf Ronneberger of the Computer Science Department and BIOSS Center for Biological Signaling Studies, the University of Freiburg, Germany. It offers the possibility to work on images in shades of gray, including the diagnostic radiological images, while the main task is the segregation and segmentation of images with potent coding and decoding solutions.

**Figure 1:**
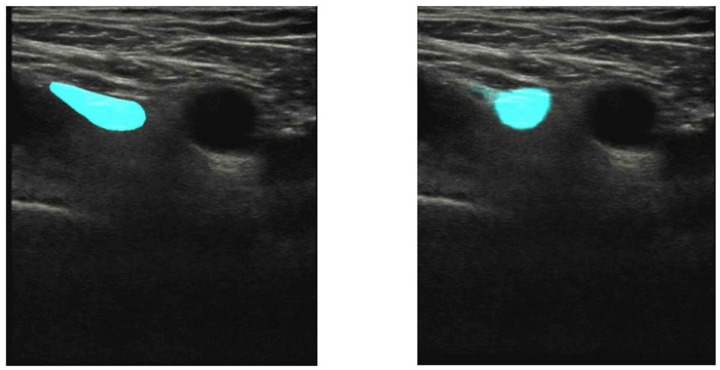
The left image shows identification of the femoral nerve (blue color) by an expert, the right one—identification via the U-Net architecture (from [10]).

**Figure 2:**
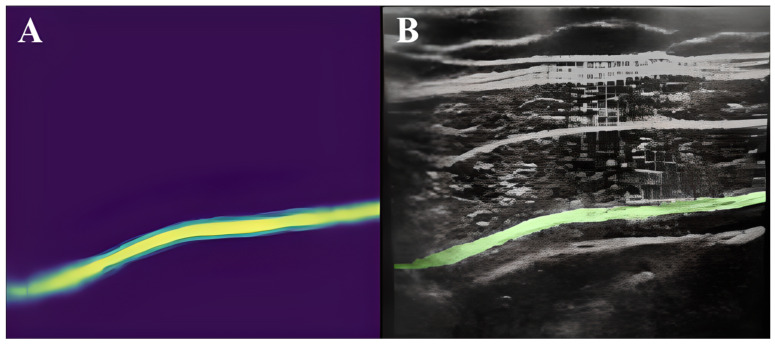
Transverse abdominis (green color) model prediction on a randomly selected image (**A**) and labeling on the ultrasound image (**B**) (from [11]).

Such a breakthrough and precise technologies capable of differentiating organs and other anatomical structures through the pixel analysis enhance the clinicist’s efficiency who assesses the diagnostic images in a classical way. U-net will precisely probe among the most important development pathways for the identification and classification of anatomical structures [14,15]. The clinical studies carried out by the Department of Experimental Surgery, McGill University, Montreal, Quebec, Canada, evaluated the usability of the U-net architecture in the context of identifying the interfascial space TAP, and those conducted in the hospital in Hässleholm, Sweden, evaluated the localization of the femoral nerve, showing that this technology may offer great support, in particular, to less experienced practitioners (Figure 1 and Figure 2).

**Figure 3:**
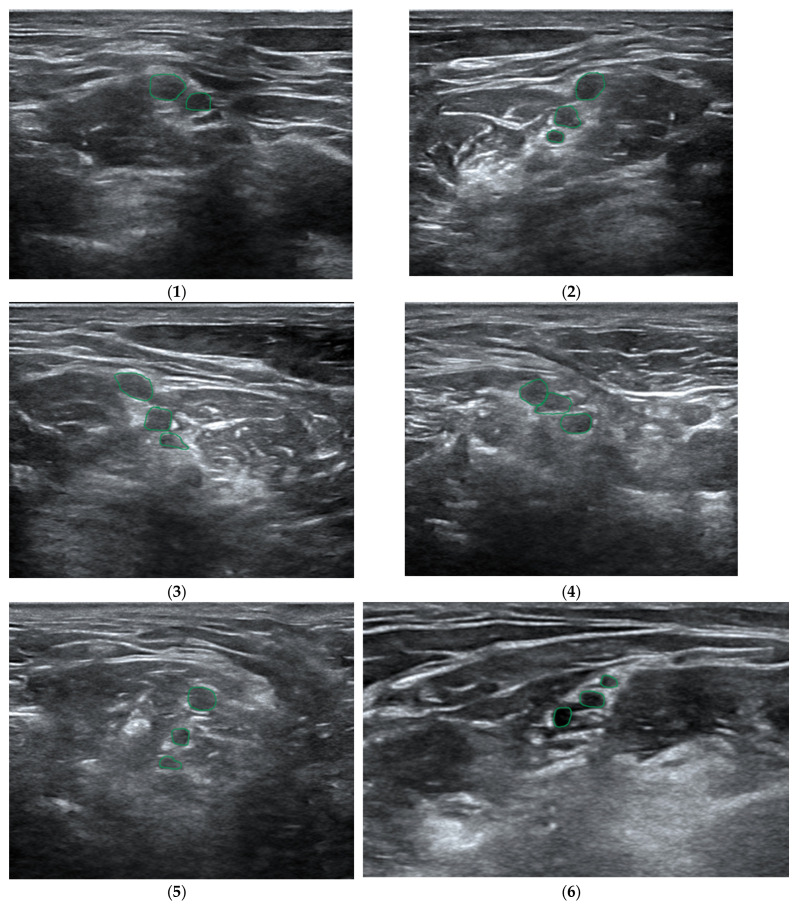
Visual presentation of segmentation for six different patients (**1**–**6**) using BPSegSys database (green color—brachial plexus).

Results from a large sample, i.e., 25,000 images for the TAP space and 1410 for the femoral nerve, obtained within the so-called big data framework, confirmed the effectiveness of the method offered by the U-Net, at the level of 73.3% and 74%, respectively [10,11]. Another study, which compared the effectiveness of the deep learning scheme with manual segmentation performed by a physician for the visualization of the brachial plexus, proved, on the basis of the gathered data (BPSegData—Figure 3), the effectiveness of the identification of that structure at the level of approximately 50% [16].

Today there are several platforms based on machine learning technology at different levels of development which may offer significant support to medical practitioners in the area of regional anesthesiology, the most important of which include the following listed below.

**Figure 4:**
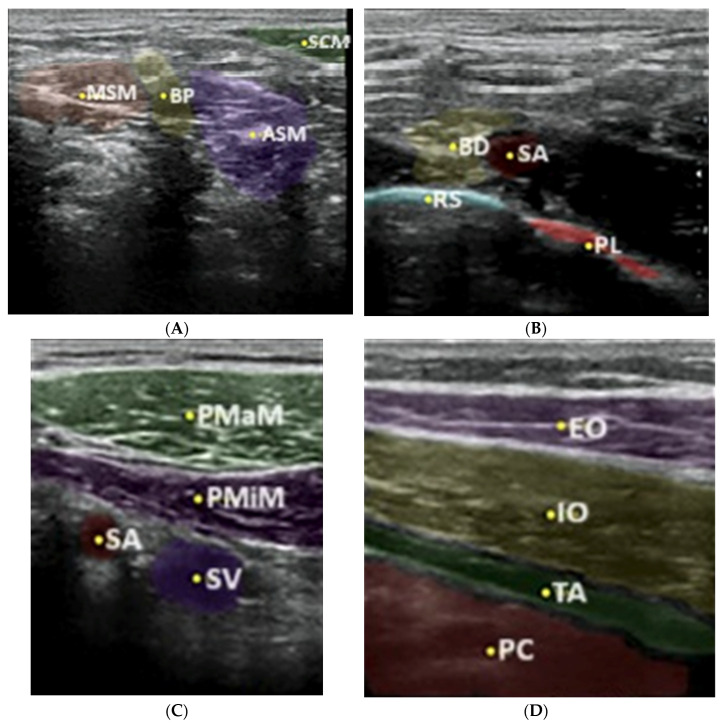
Boundary prediction of interscalene block (**A**): SCM sternocleidomastoid muscle, ASM anterior scalene muscle, BP brachial plexus, MSM middle scalene muscle; supraclavicular block (**B**): BP brachial plexus, SA subclavian artery, RS rip shadow, PL parietal pleura; infraclavicular block (**C**): PMaM pectoral major muscle, PMiM pectoral minor muscle, SV subclavian vein, SA subclavian artery and TAP block (**D**): EO external oblique, IO internal oblique, TA transversus abdominis, PC peritoneal cavity with predefined anatomic landmarks in color-labeled overlay (from [6]).

Nerveblox—a technology supplied by Smart Alfa Teknoloji San. Ve Tic. A.S., Ankara, Turkey, is a software implemented in 2020 to convert the ultrasound images downloaded in real time from the ultrasound system to the computer, presenting then the anatomical structures, e.g., muscles, pleura, arterial and venous vessels, bone structures and, of course, nerves and nerve plexuses in the given colors (Figure 4). 

**Figure 5:**
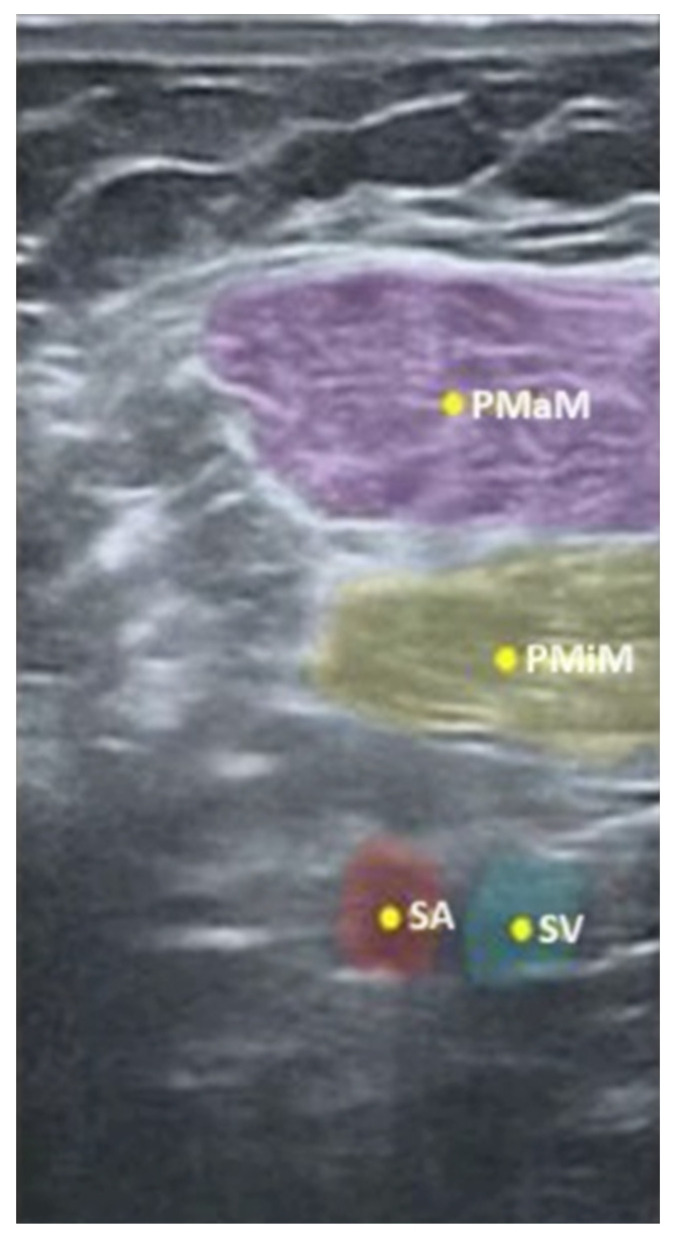
Nerveblox image of the four anatomical regions defined for infraclavicular block (PMaM pectoralis major muscle, PMiM pectoralis minor muscle, SA subclavian artery, SV subclavian vein). (From [17]).

Another example to prove the proper prediction of a particular anatomical structure is an USG image, presented in Figure 5, reflecting the pectoralis major, the pectoralis minor as well as the subclavian artery and vein.

**Figure 6:**
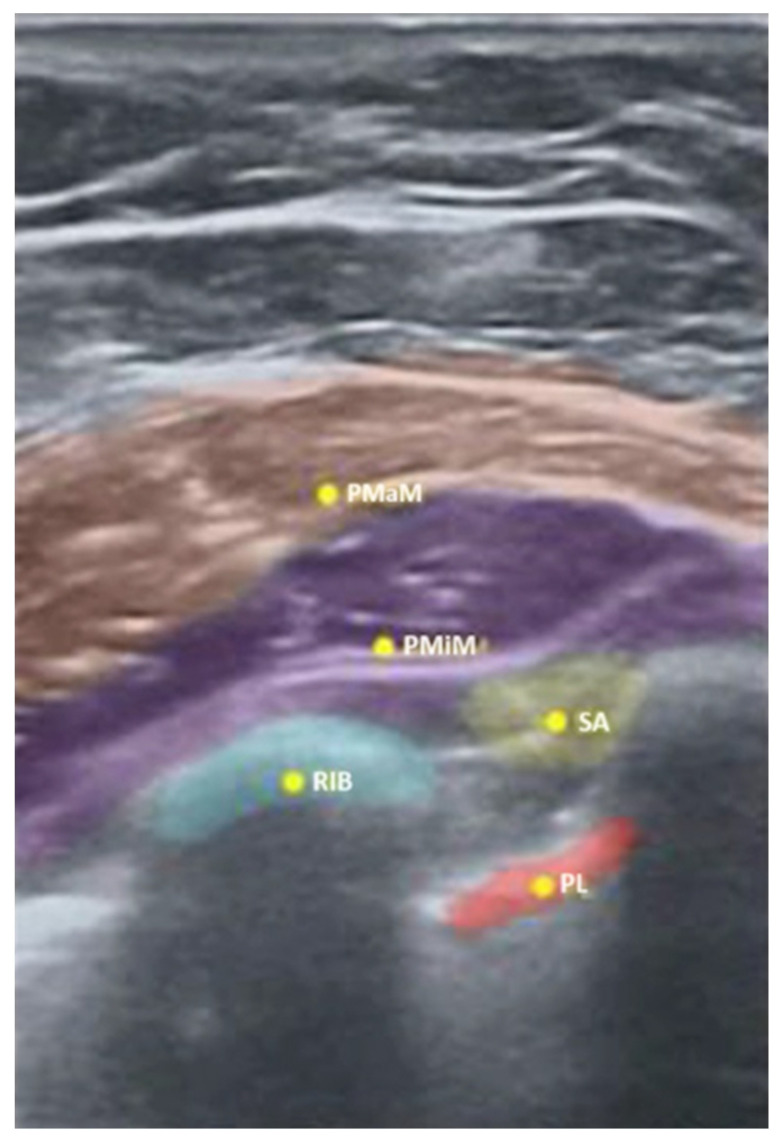
Nerveblox image of five anatomical regions determined for PECS I and II blocks (PMaM pectoralis major muscle, PMiM pectoralis minor muscle, SA serratus anterior muscle, PL parietal pleura, RIB first rib). (From [17]).

As illustrated in Figure 5, the Nerveblox technology may also be used in regional anesthesia to identify the interfascial space, while Figure 6 below indicates, in yellow, the spots to deposit the local anesthetic for PECS I and II blocks.

**Figure 7:**
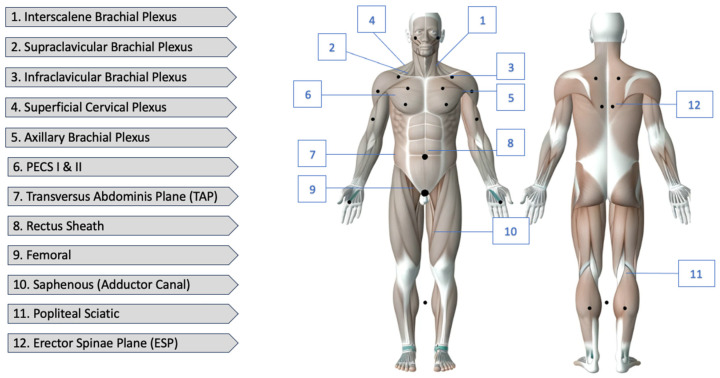
Nerveblox supports the following peripheral nerve block (PNB) types.

The Nerveblox is capable of recognizing the USG images in real time thanks to the abovementioned mathematical algorithms referred to as the convolutional neural networks (ConvNets). The potential of neural networks is immense, yet their capabilities are now used on a marginal scale. The technology itself is based on the detection of highly complicated relations between the captured images and those stored by the device (Nerveblox, in this very case), as well as the correlation between the adjacent pixels. In the case of accordance, the computer automatically confirms the case and assigns particular colors to the individual anatomical structures [18,19]. At present, Nerveblox is dedicated to 12 regional blocks, the details of which are illustrated in Figure 7. 

**Figure 8:**
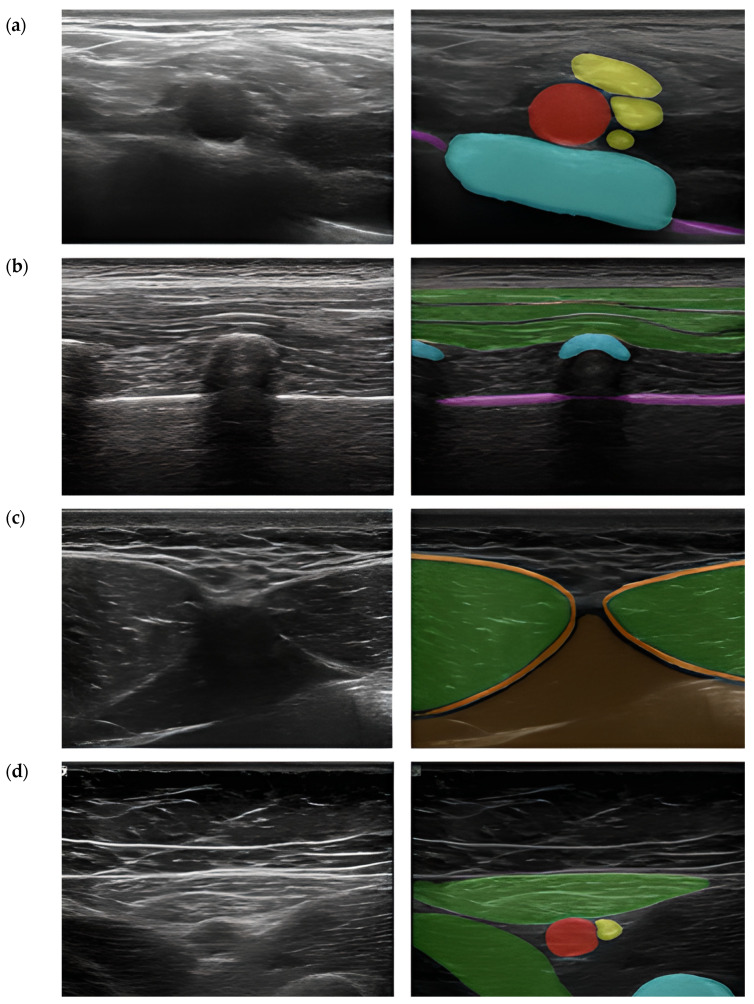
Still images taken from ultrasound videos labeled by ScanNav Anatomy PNB. (**a**) Supraclavicular-level brachial plexus: subclavian artery (red), brachial plexus nerves (yellow), first rib (blue), pleura (purple). (**b**) Erector spinae plane (thoracic region): trapezius/rhomboid/erector spinae (group) muscles (green), vertebral transverse process/rib (blue), pleura (purple). (**c**) Rectus sheath: rectus abdominis muscle (green), rectus sheath (orange), peritoneal contents (brown). (**d**) Adductor canal: femoral artery (red), saphenous nerve (yellow), sartorius/adductor longus (green), femur (blue) (From [20]).

ScanNav Anatomy Peripheral Nerve Block and NeedleTrainer (software version 2.2) is a system supplied by Intelligent Ultrasound, Cardiff, UK. Also supported by artificial intelligence while scanning the given regions of the patient’s body, it creates a colorful overlay on the monitor to indicate the requested anatomical structures. Similar to Nerveblox, the device makes use of deep learning based on the U-Net architecture. Here, the so-called big data is a database containing 800,000 models of USG images which make the points of reference for particular anatomical structures, in consequence creating a colorful overlay, as presented in Figure 8 [15,20,21].

**Figure 9:**
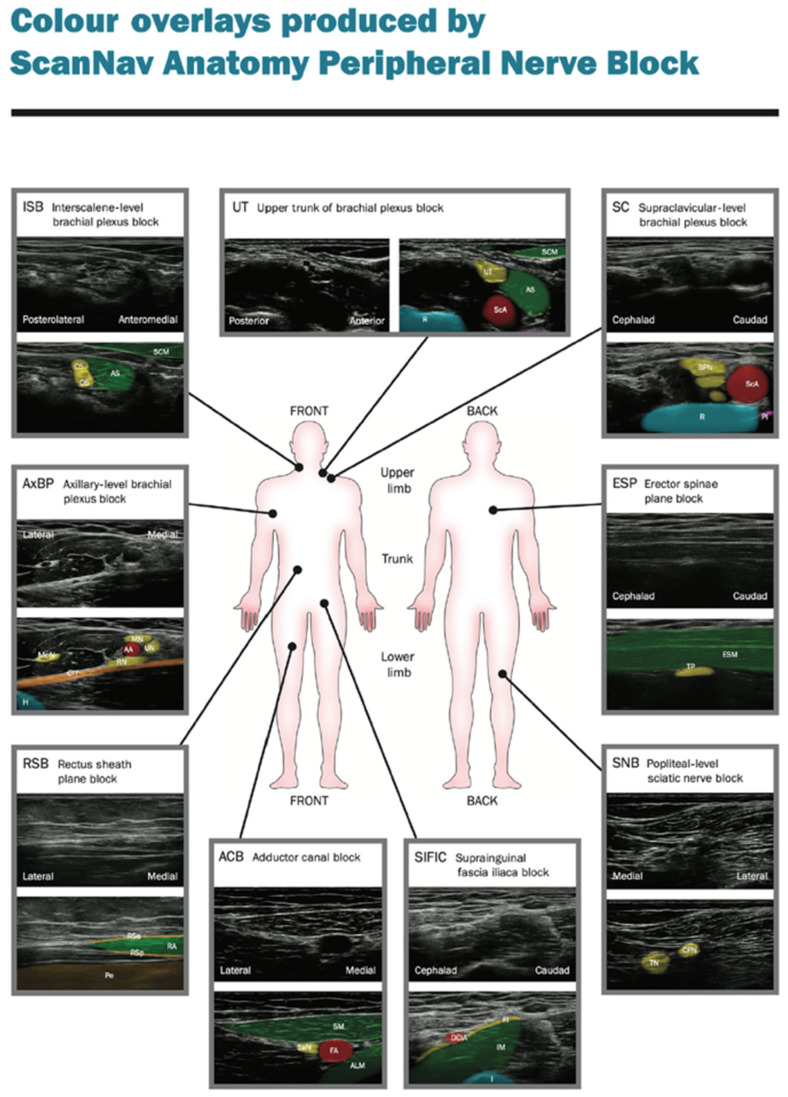
Examples of artificial intelligence color overlay for each peripheral nerve block studied (from [21]).

ScanNav Anatomy PNB presents the ten most common types of regional block (Figure 9). They are the following:

- Axillary-level brachial plexus;
- Erector spinae plane;
- Interscalene-level brachial plexus;
- Popliteal-level sciatic nerve;
- Rectus sheath plane;
- Sub-sartorial femoral triangle/adductor canal;
- Superior trunk of brachial plexus;
- Supraclavicular-level brachial plexus;
- Longitudinal suprainguinal fascia iliaca plane.

The appropriate selection of a regional block is confirmed by the fact that 7 out of 10 examples specified in the so-called Plan A Regional Anesthesiology (the basic teaching standard—Table 1) are contained in the ScanNav Anatomy software.

It is also worth mentioning that the system cooperates with classical USG system devices furnished with digital sockets to transmit images—HDMI or DVI. Note also that in October 2022, ScanNav Anatomy was approved by the American Food and Drug Administration (FDA). The system has been qualified within the framework of a de novo program which effectuated the formation of a new category of devices for the visualization and color coding of anatomical structures used in AI-supported regional anesthesia [22,23].

**Figure 10:**
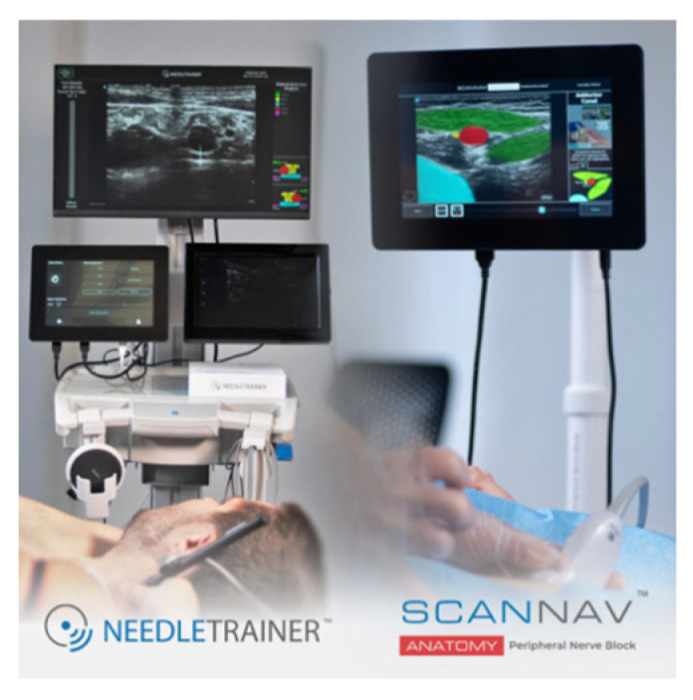
SkanNav Anatomy presenting color-coded anatomy along with an instruction video (from: https://www.intelligentultrasound.com/scannav-anatomy-pnb accessed on 1 January 2024).

Apart from the color-coded identification of anatomical structures, the system is also capable of simultaneous presentations of instruction videos showing techniques used to accomplish a selected procedure of regional anesthesia which makes it an excellent training tool for any anesthesiologist performing regional anesthesia (Figure 10).

**Figure 11:**
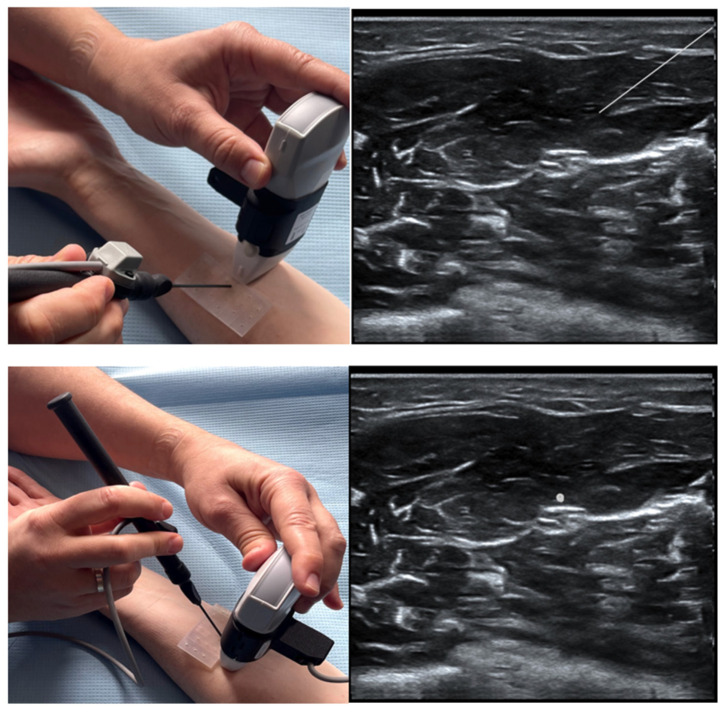
Image of a simulated rectus sheath block using the NeedleTrainer™ hardware. Left image: NeedleTrainer™ transducer and needle. Right image: simulated needle on real-time ultrasound image.

The latest function furnishing ScanNav Anatomy is the NeedleTrainer which makes use of retractable needles for an instant invasive simulation of needling in human tissue. The major advantage of this solution is the possibility of developing the skills, in particular “hand–eye” coordination, during any invasive procedures including the regional block (Figure 11). However, the technology itself is not a haptic solution and does not fully reflect the block condition, appearing as a challenge for manufacturers [24].

The clinical studies carried out so far with the use of ScanNav Anatomy have clearly justified the application of the technology to support trainees as well as expert educators in the area of regional anesthesia [25,26]. This thesis may be confirmed by the results collected and described in April 2021, following a clinical study carried out in several British hospitals. During the study, experts were asked a number of questions. The study made use of the Delphi surveying method where experts highly valued the ability of ScanNav to highlight the anatomical structures of key importance for the block, and the highest scores where achieved for imaging the rea of the adductor canal, the brachial plexus in the area of the axillary fossa and the iliac fascia superior to the inguinal ligament. In the experts’ opinion, the applicability of the software to recognize individual critical structures to accomplish a technically correct block ranged between 95% and 100%, while highlighting the localization target was assessed at the level of 100% in the case of 31 anatomical structures out of 34 evaluated ones. The experts emphasized that in five cases out of the seven evaluated areas (i.e., the plane of the spinal extensor, the rectus abdominis sheath, the iliac fascia above the inguinal ligament, the adductor canal and the sciatic nerve in the area of the popliteal fossa), ScanNav would ensure a 100% benefit for less experienced practitioners and the medical trainees when confirming the correct location and positioning of the ultrasound head, while in the remaining two areas (imaging of the brachial plexus in the axillary fossa and the supraclavicular region), the score amounted to 97.5% [20].

### 2.2. Artificial Intelligence Used Directly in the Ultrasound Systems

**Figure 12:**
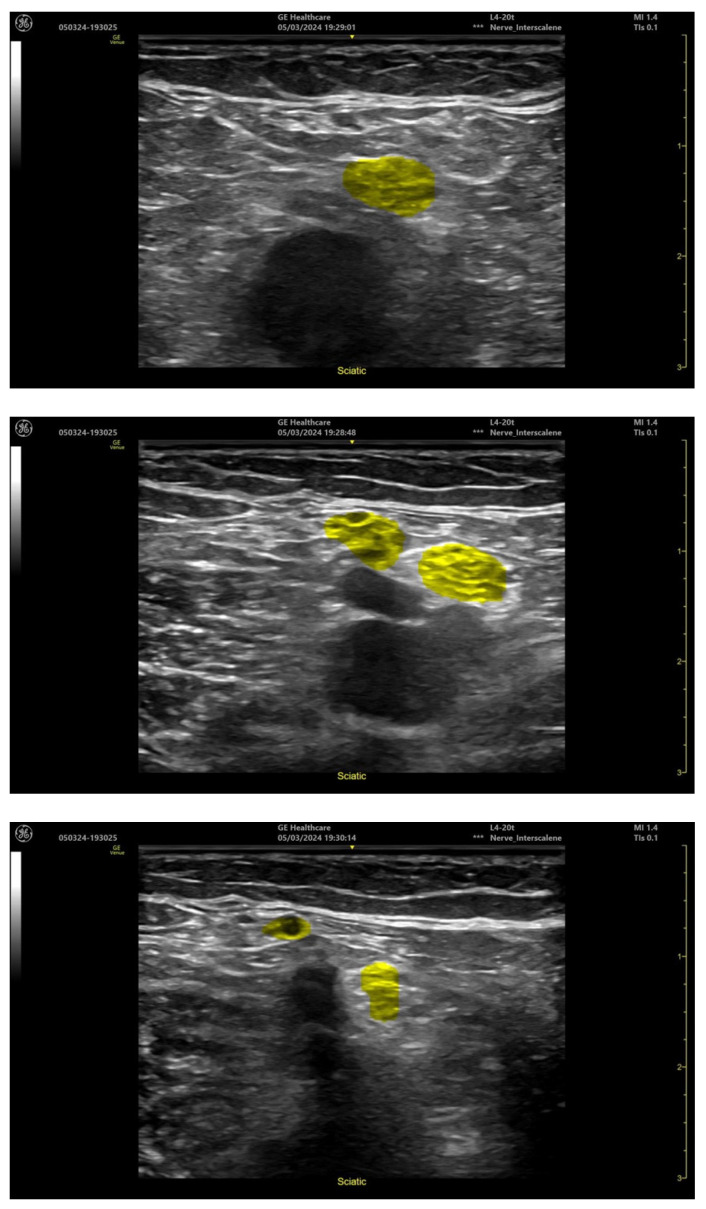
Popliteal sciatic nerve block location (GE Venue Fit, software version 303.45.0.345).

**Figure 13:**
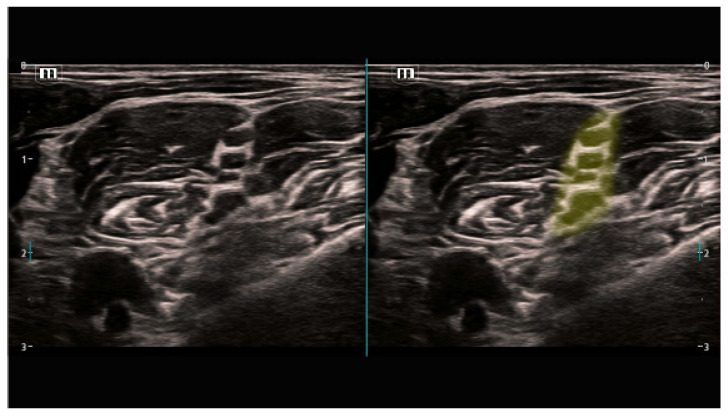
Brachial plexus (supraclavicular) nerve block location (Mindray TEX20, software version 01(01.07.00)).

**Figure 14:**
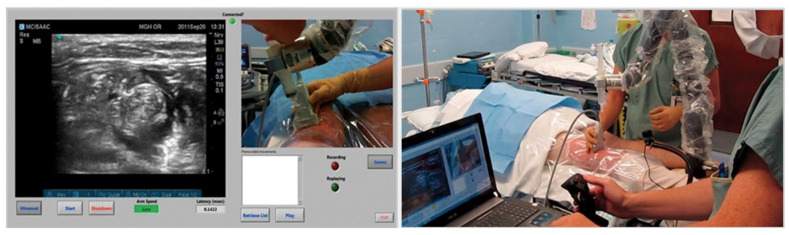
Main components of Magellan robot.

Manufacturers of the ultrasound systems have already recognized the need for the implantation of state-of-the-art AI technologies in their devices. In order to meet the users’ expectations and to address the medical practitioners’ constantly growing interest in new technologies, such manufacturers as Mindray Medical International Limited or GE HealthCare have introduced such solutions to their system, already without any accessory devices such as Nerveblox or ScanNav Anatomy [27,28]. Of special interest are Mindray SmartNerve or GE cNerve technologies whose capacities are illustrated in the Figure 12 and Figure 13 below [29,30]:

Magellan, on the other hand, is a semi-automatic, robotized system used to perform USG-guided nerve blocks with the use of a remote control center produced by Oceanic Medical Products, Inc., Atchison, KS, USA. Magellan incorporates three major elements, including the joystick, the robot arm and the computer with the procedure control software, as illustrated in Figure 14.

Using the joystick, the operator controls the robot arm with the syringe and the needle at three different speeds. The fastest is used to shift the arm into the area of the regional anesthesia, the medium one to introduce the needle into the tissue and the slowest one when approaching a particular nerve, plexus or the interfascial space. Unfortunately, the identification of the nerve itself is still performed in a classical way, i.e., by a man, while the remaining procedures are carried out by a robot controlled remotely by an operator. Some convenience in identifying the nerves is a solution which allows for the reduction in the artefacts with simultaneously improved echogenicity of the evaluated neural structure. Visual improvement is accomplished by the control panel. Two handles of the joystick ensure movements similar to those of a human wrist and arm while performing manually the block procedures. A practical test of the technology comprising 13 patients and 16 block procedures showed no complications; each of the blocks proved effective, and the average time of the procedure was 3 min [1,31,32].

## 3. Challenges, Limitations and Evaluation of Effectivity of AI Applications in Ultrasonography and Anesthesiology

So far, the clinical studies related to ScanNav Anatomy have explicitly justified the use of this system as a supporting solution for beginners as well as experts educating in the area of regional anesthesiology. The thesis may be proved by the results collected and described in April 2021 on the basis of a clinical study carried out in several British hospitals. The experts were asked a series of questions, and the selected answers are presented in Table 2, Table 3 and Table 4. The study made use of the Delphi survey method [15,26].

1. Does the video contain clinically relevant images for this block area? [Y/N].

**Table 2:** A summary of the overall highlighting performance for each block area.

	NeckBP	AxBP	ESP	RS	FI	AC	PopSN
**Min**	5.33	5.33	6.33	5.67	6.33	5.67	5.67
**Max**	9.33	10.00	9.67	9.00	10.00	9.67	9.33
**St Dev**	1.017	0.981	0.666	0.816	0.812	0.698	0.867
**Mean**	7.89	8.43	8.10	7.87	8.42	8.69	8.09

Abbreviations: NeckBP: interscalene-supraclavicular level brachial plexus; Ax—axillary level brachial plexus; ESP: erector spinae plane; FI: suprainguinal fascia iliaca; RS: rectus sheath; AC: adductor canal; PopSN: popliteal level sciatic nerve.

2. Rate the overall highlighting performance on a scale of 0–10. [0—very poor, 10—excellent].

**Table 3:** Expert opinion on whether highlighting helped to identify individual structures.

Structure	Yes	No
Interscalene- Supraclaviular Level Brachial Plexus		
Subclavian artery	40/40 (100%)	0/40 (0%)
Brachial plexus nerves	40/40 (100%)	0/40 (0%)
Sternocleidomastoid muscle	40/40 (100%)	0/40 (0%)
Scalenus anterior muscle	40/40 (100%)	0/40 (0%)
First rib	40/40 (100%)	0/40 (0%)
Pleura	40/40 (100%)	0/40 (0%)
Total (for block)	240/240 (100%)	0/240 (0%)
Axillary Level Brachial Plexus		
Axillary artery	40/40 (100%)	0/40 (0%)
Radial nerve	40/40 (100%)	0/40 (0%)
Median nerve	40/40 (100%)	0/40 (0%)
Ulnar nerve	40/40 (100%)	0/40 (0%)
Musculocutaneous nerve	38/40 (95%)	2/40 (5%)
Fascia (conjoint tendon)	40/40 (100%)	0/40 (0%)
Humerus	40/40 (100%)	0/40 (0%)
Total (for block)	278/280 (99.3%)	2/280 (0.7%)
Erector Spinae Plane		
Muscle layer (Trapezius, rhomboid, erector spinae)	35/35(100%)	0/35 (0%)
Ribs	35/35(100%)	0/35 (0%)
Transverse process	35/35(100%)	0/35 (0%)
Pleura	35/35(100%)	0/35 (0%)
Total (for block)	140/140(100%)	0/140(0%)
Rectus Sheath		
Restus abdominis muscle	40/40 (100%)	0/40 (0%)
Transversus abdominis muscle	40/40 (100%)	0/40 (0%)
Rectus sheath	40/40 (100%)	0/40 (0%)
Peritoneum/peritoneal contents	40/40 (100%)	0/40 (0%)
Total (for block)	160/160 (100%)	0/160 (0%)
Suprainguinal Fascia Iliaca		
Deep circumflex iliac artery	37/38 (97.4%)	1/38 (2.6%)
Iliacus muscle	40/40 (100%)	0/40 (0%)
Fascia iliaca	40/40 (100%)	0/40 (0%)
Hip bone	40/40 (100%)	0/40 (0%)
Total (for block)	157/158 (99.4%)	1/158 (0.6%)
Adductor Canal		
Femoral artery	40/40 (100%)	0/40 (0%)
Saphenous nerve	40/40 (100%)	0/40 (0%)
Sartorius muscle	40/40 (100%)	0/40 (0%)
Adductor longus muscle	40/40 (100%)	0/40 (0%)
Femur	38/38 (100%)	0/38 (0%)
Total (for block)	198/198 (100%)	0/198(0%)
Popliteal Level Sciatic Nerve		
Popliteal artery	39/40 (97.5%)	1/40 (2.5%)
Sciatic nerve	40/40 (100%)	0/40 (0%)
Tibial nerve	39/39 (100%)	0/39(0%)

3. Did the highlighting help identify the [insert structure name]? [Y/N].

**Table 4:** Expert opinion on whether highlighting would help confirm the correct ultrasound view to a less experienced practitioner.

	NeckBP	AxBP	ESP	RS	FI	AC	PopSN
**Y (%)**	39/40 (97.5%)	39/40 (97.5%)	35/35 (100%)	40/40 (100%)	40/40 (100%)	40/40 (100%)	40/40 (100%)
**N (%)**	1/40 (2.5%)	1/40 (2.5%)	0/35 (0%)	0/40 (0%)	0/40 (0%)	0/40 (0%)	0/40 (0%)

Abbreviations: NeckBP: interscalene-supraclavicular level brachial plexus; Ax: axillary level brachial plexus; ESP: erector spinae plane; FI: suprainguinal fascia iliaca; RS: rectus sheath; AC: adductor canal; PopSN: popliteal level sciatic nerve.

Also, another study which aimed to assess the validity of AI in ultrasonography justified the use of such technology for the interpretation of anatomical structures. The investigations comprised Nerveblox technology where the scheme one to five points (five = the highest score) was used to evaluate the reliability of the sonoanatomical structures indicated by AI. The details and results of the study are presented in the Table 5 below [6].

The evaluation of the use of ScanNav Anatomy was also the objective of a study, published in September 2022 in the British Journal of Anesthetic, which comprised 126 procedures performed by 21 anesthesiologists, none of whom were experts in peripheral block. The Table 6 below confirm again that the use of AI brings substantial improvement in the interpretation of the obtained sonoanatomical images [21].

**Figure 15:**
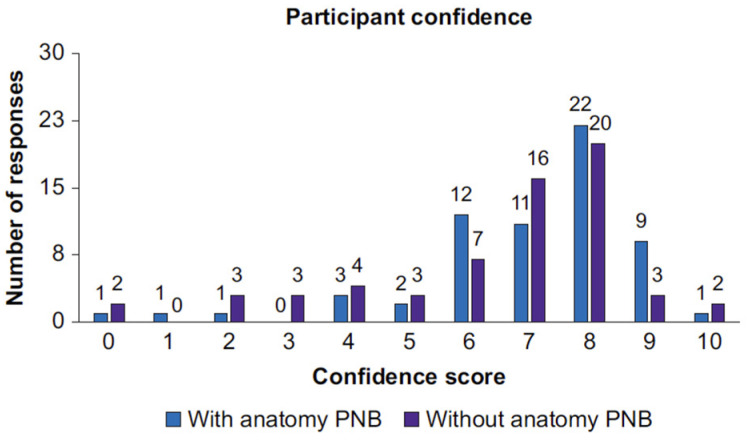
Participant confidence score. Distribution of all participant self-rated confidence scores, showing a breakdown of scans performed with or without ScanNav Anatomy peripheral nerve block. PNB, peripheral nerve block.

**Figure 16:**
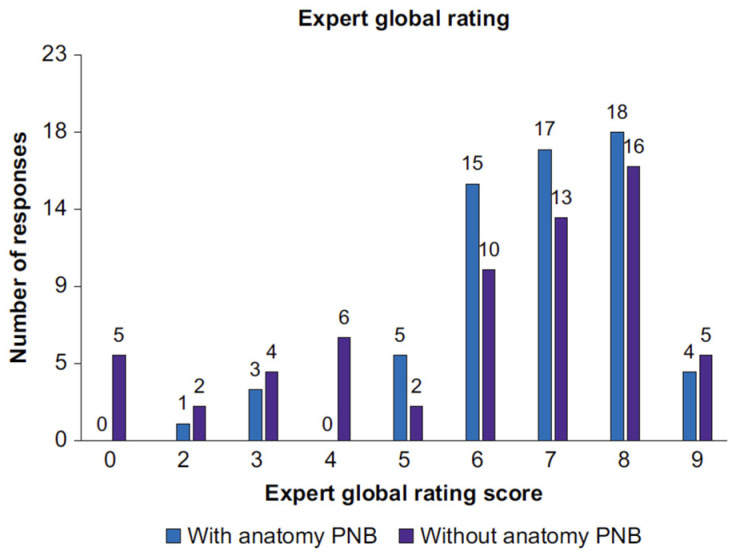
Expert global rating score. Distribution of all expert global rating scores, showing a breakdown of scans performed with or without ScanNav Anatomy peripheral nerve block. PNB, peripheral nerve block.

AI shows an immense potential, enough to revolutionize the whole of the healthcare system, first of all improving its effectivity which may be preceded by the assistance of training programs and education. Innovative as it is, the technology is capable of stimulating comprehensive developments in the area of anesthesiology. The applications in anesthesiology include monitoring life parameters, predicting adverse occurrences, adjustment of the medication dosage and automated storage of medical records. The progress brought to anesthesiology by AI, starting from early expert systems to reach up-to-date advanced technologies, shows AI’s power to transform the healthcare system (Figure 15 and Figure 16).

Nevertheless, the implementation of AI in anesthesiology is associated with multiple challenges. These include the questions of quantity and quality of the data as well as their validation, some technological limitations and finally the ethical and legal issues. AI systems may enhance prejudice should they be based on faulty data. Oppressed privacy appears as another threat when appropriate safety measures are not assumed. High cost may be an obstacle to introduce it on a daily basis. Moreover, there still remains a question of legal responsibility in case an AI system error puts the patient’s health and life at risk. The user’s willingness to use AI in daily practice is also affected by interface solutions and the working environment; they should be user-friendly and clear to understand, any problems easy to solve and routines preceded by appropriate implementation procedures and training programs [33].

It is expected that future roles of AI in anesthesiology will be growing to bring significant changes and to open new areas of application. Among the most marked tendencies is the further development of AI and of machine learning. Such technologies are becoming even more precise, reliable and capable of solving more complex problems. An example is deep learning where neuron networks are used to analyze massive amounts of data and find their complex relations. This may have a great role in anesthesia ensuring better predictions of the result and the risk of complications. Recent investigations focus also on potential novel applications of AI in anesthesiology, e.g., in pain management. AI systems are capable of analyzing the physiological data reported by patients to evaluate the level of pain and administer proper analgesics. Another area includes therapies of chronic diseases demanding anesthesia, for example, chronic pain management or palliative care. AI may assess data from the long-term perspective and deliver information about the effectivity of the applied anesthetic procedures, supporting, in this way, correct therapeutic decisions.

AI may also revolutionize education and training schemes in the area of anesthesiology. It would be also possible to create realistic simulations, an opportunity for the students to improve their skills in safe conditions. A system may also provide tailored personal feedback pointing to the strong and weak points and offering specific suggestions for self-development.

Artificial intelligence could also assist the continuous learning of anesthesiologists, analyze data of their daily practice and indicate those areas which need improved skills and competence.

Nevertheless, it should be remembered that artificial intelligence makes no replacement for an anesthesiological practitioner; human intervention and expertise will remain indispensable. However, AI may enhance the anesthesiologists’ potential, offering support and assistance while making more conscious choices and evaluating situations.

Recommendations for future investigations and development in the area of artificial intelligence in anesthesiology are as follows:

1. Security and the role of data:
  - Further training programs and new procedures are required for data security, collection and processing.
  - Respective provisions of the law regarding data processing should be prepared.
2. Accuracy and comprehension of AI systems:
  - Further studies on the accuracy and comprehension of artificial intelligence systems should be carried out.
  - Clarity and precision of the AI algorithms need to be improved.
3. Influence of artificial intelligence on teaching and training in anesthesiology:
  - Investigations should be continued to recognize the influence of artificial intelligence on teaching and training in anesthesiology.
  - AI-based simulation programs should be designed.
  - A non-standard feedback system based on artificial intelligence should be developed.
  - It should be investigated how simulation programs and feedback systems based on artificial intelligence improve the learning outcome [33].

Although the way to common implementations of artificial intelligence may be long and windy, its potential is just enormous.

## 4. Conclusions

Artificial intelligence has revolutionized medicine and became a tool significantly facilitating the identification of anatomical structures, taking measurements and reducing the duration of medical procedures, at the same time making it easier for less skilled practitioners to perform proper ultrasound imaging. Will artificial intelligence replace physicians in the area of regional anesthesia? This would require a fully autonomous robot capable of making decisions on its own.

Taking into consideration the ethical and legal aspects, such solutions seem unlikely. However, the near future will bring systems instructing an operator precisely on how to image individual neural and perineural structures, showing the needling axis and angle, the volume of the local anesthetic and detecting improper, e.g., intramuscular, administration of a drug. This will result in a reduced number of block attempts, fewer complications and a smaller burden for medical personnel.

## Figures and Tables

**Table 1:** Proposed high value basic ultrasound-guided regional anesthetic techniques.

Anatomical Location	Plan A (Basic Blocks)	Plan B/C/D (Advanced Blocks)
Upper limb		
Shoulder	Interscalene brachial plexus block [14]	Superior trunk block, combined axillary and suprascapular nerve blocks
Below shoulder	Axillary brachial plexus block [15]	Infraclavicular block, supraclavicular block
Lower limb		
Hip	Femoral nerve block [16]	Fascia iliaca block, lumbar plexus block
Knee	Adductor canal block [17]	Femoral nerve block ± IPACK block
Foot and ankle	Popliteal sciatic block [18]	Ankle blocks, proximal sciatic nerve block
Trunk		
Chest wall	Erector spinae plane block [19]	Paravertebral block, serratus plane block, PECS blocks
Abdominal midline	Rectus sheath block [20]	Quadratus lumborum blocks

IPACK, interspace between the popliteal artery and the capsule of the posteriori knee; PECS, pectoral nerves. Refers to selective blocks from the distal femoral triangle to Hunter’s canal.

**Table 5:** Representation of landmark labels of block types and assessment of each validator (V) according to selected block-associated anatomical landmarks (means +/− d).

Block Type	Predefined Anatomaical Landmarks	V1	V2	*p*
Interscalene	Brachial plexus (BP)	4.84 ± 0.47	4.92 ± 0.41	0.96
	Anterior scalene muscle (ASM)	4.89 ± 0.31	4.87 ± 0.33	0.98
	Middle scalene muscle (MSM)	4.88 ± 0.37	4.86 ± 0.35	0.95
	Sternoleidomastoid muscle (SCM)	4.96 ± 0.2	4.94 ± 0.22	0.96
Supraclavicular	First rib (FR)	5 ± 0.01	4.98 ± 0.12	0.96
	Pleura (PL)	5 ± 0.01	4.98 ± 0.12	0.96
	Subclavian artery (SA)	5 ± 0.01	4.98 ± 0.05	0.97
	Brachial plexus (BP)	4.9 ± 0.01	4.98 ± 0.04	0.99
Infraclavicular	Pectoralis major muscle (PMJ)	5 ± 0.01	4.97 ± 0.08	0.95
	Pectoralis minor muscle (PMN)	5 ± 0.01	4.98 ± 0.07	0.96
	Axillary artery (AA)	5 ± 0.01	4.99 ± 0.05	0.99
	Axillary vein (AV)	4.39 ± 0.26	4.95 ± 0.12	0.94
TAP	Transverse abdominis muscle (TAM)	5 ± 0.01	4.98 ± 0.1	0.98
	Internal oblique muscle (IOM)	4.98 ± 0.16	4.97 ± 0.12	0.96
	External oblique muscle (EOM)	4.98 ± 0.16	4.96 ± 0.15	0.92
	Peritoneal cavity (PC)	4.95 ± 0.22	4.93 ± 0.2	0.95

**Table 6:** Summary of overall endpoints. AI, artificial intelligence; IQR, inter-quartile range; SD, standard deviation.

	Scanning with AL Assistive Device	Scanning without AL Assistive Device	Alpha (p-Value)
Correct block view, *n* (%)	56/62 (90.3)	47/62 (75.1)	0.031
Correct structure identification, *n* (%)	188/212 (88.8)	161/208 (77.4)	0.002
Median confidence (IQR)	8 (6–10)	7 (6–10)	0.155
Median global rating score (IQR)	7 (6–9)	7 (4–9)	0.225
Mean scan time (SD), s	75.9 (69.6)	74.5 (65.6)	0.881

## Data Availability

Not applicable.
